# The role of video-based discussion in model for preparing professional development leaders

**DOI:** 10.1186/s40594-017-0090-3

**Published:** 2017-11-22

**Authors:** Hilda Borko, Janet Carlson, Charmaine Mangram, Robin Anderson, Alissa Fong, Susan Million, Suki Mozenter, Anthony Muro Villa

**Affiliations:** Stanford University, Stanford, USA

**Keywords:** Teacher leadership, Math education, Video-based discussion, DBIR, Teacher professional development

## Abstract

**Background:**

This paper describes the Problem-Solving Cycle model of professional development and the Mathematics Leadership Preparation model of PD leader preparation. These models form the backbone of our current research-practice partnership project in which we are working with a large urban district to adapt these models to develop district capacity to support the implementation of a middle school mathematics curriculum aligned with Common Core State Standards (CCSS). We highlight the central role of video in the Problem-Solving Cycle and our approach to preparing teacher leaders to use video-based discussions to understand student thinking and instructional practices.

**Results:**

The first phase of the research was designed to identify how the models were adapted to support the district goals for implementing their new CCSS mathematics curriculum and to understand the reasons for the adaptations. The analysis of multiple data sources revealed two overarching categories of adaptations that we made to refine the models to better support the district goals: addressing district priorities and addressing teacher leaders’ limited experience. We made adaptations such as incorporating the district curriculum, addressing the needs of English learners, integrating the teacher leaders’ learning of the Problem-Solving Cycle model into the leadership preparation session, increasing the emphasis on what it means to be an instructional leader, strengthening the role of modeling and debriefing activities to support leadership development, scaffolding the selection of video clips, and incorporating the use of rehearsals and debriefing activities to support leadership development.

**Conclusion:**

The implications of this work illustrate the need for researchers to be responsive to the context of their school partners if they expect their work to be meaningful. Using the frame of design-based implementation research proved to be an effective strategy for working with the district STEM leadership team and teacher leaders to adapt the Problem-Solving Cycle and Mathematics Leadership Preparation models to support district implementation of a new curriculum that necessitates shifts in teacher practices and for determining how to make video-based discussions more productive activities in the models.

## Background

In recent years, there has been an increasing demand for professional development for K-12 teachers. This need is particularly strong in mathematics, given the combination of teachers’ and students’ inadequate knowledge bases and high demand for young people to be prepared for careers in STEM disciplines (Organization for Economic Cooperation and Development [OECD], 2007). In the USA, this demand has been heightened by widespread adoption of the Common Core State Standards (CCSS). “Developing teachers’ capacity to enact new standards in ways that support the intended student learning outcomes will require considerable changes in mathematics instruction in our nation’s classrooms. Such changes are likely to occur only through sustained and focused professional development opportunities for those who teach mathematics” (Marrongelle et al. 2013, p. 208). A central feature of professional development (PD) programs that are scalable and sustainable is the preparation of leaders who can implement the program with integrity, adapting it to local contexts while maintaining consistency with core principles (Borko et al. 2011).

Practicing mathematics teachers are one obvious personnel source to assume leadership positions, especially for site-based PD. For teachers, however, working on mathematics learning and instruction with adult learners—typically colleagues in their schools—is very different than their usual work of teaching mathematics to K-12 students. How can we foster the capacity of a large base of mathematics teachers to become PD leaders in their schools? What guidance and support do novice facilitators need to be successful? Researchers are just beginning to investigate these questions (e.g., Borko et al. 2014; Elliott et al. 2009; Jackson et al. 2015; Lesseig et al. 2016).

This paper describes the Problem-Solving Cycle (PSC) model of professional development, the Mathematics Leadership Preparation (MLP) model of PD leader preparation (Borko et al. 2015), and our current research project to adapt these models for implementation in the Urban Unified School District (UUSD)^1^. We highlight the adaptations we made to keep the role of video central in the PSC and to strengthen our approach to preparing teacher leaders to lead video-based discussions (VBDs). In the original models, teacher leaders engage their colleagues in a Problem-Solving Cycle (PSC) using a common math task and collecting video of their own teaching. As a result of our collaboration with the school district, we made several adjustments to the models during the pilot phase. These adaptations were influenced by both district priorities and the limited experience of the teacher leaders.

The project is one program of research in the Stanford University-UUSD Partnership, a *research*-*practice partnership* (RPP) that matches researchers from Stanford University’s Graduate School of Education with UUSD district leaders to solve key problems of practice. Like other RPPs, the Partnership is a long-term collaboration between practitioners and researchers that is organized to investigate persistent problems of practice and design solutions to improve educational outcomes. It includes multiple research projects that are jointly negotiated and implemented with shared authority (Coburn and Penuel 2016). The specific problem of practice we are addressing is building capacity within UUSD to conduct school-based professional development to support teachers’ implementation of their new task-based mathematics curriculum. We used the PSC and Teacher Leadership Preparation (TLP) models as the starting place for addressing this persistent problem of practice.

Our project uses a *design*-*based implementation research* (DBIR) approach to develop, adapt, and study a pair of interrelated professional development models and resources. In DBIR work, partners focus on persistent problems of practice, define shared goals, and establish a commitment to study the process of designing, adapting, and implementing the intervention so it provides mutual benefits (Fishman et al. 2013). Researchers and practitioners engage in an iterative, collaborative design process. They incorporate issues and findings emerging from implementation, making adaptations early in the program so they can be used to improve the quality of the intervention (Penuel and Fishman 2012). In this article, we report on our first cycle of design, the adaptations we made early in response to district needs, and the revisions we made during implementation. We refer to this cycle as the Design Phase of the project. The research questions we address are the following:

1. How were the Problem-Solving Cycle model of professional development and Mathematics Leadership Preparation model of PD leader preparation adapted to support the Urban Unified School District goals for implementing their new CCSS mathematics curriculum?
2. What is the nature of the adaptations made to the models?
  - How did district conditions shape the adaptations we made?
  - How did the characteristics of the teacher leaders influence the adaptations we made?

### Original models, emphasizing the role of VBD

In this section, we describe the original Problem-Solving Cycle and Mathematics Leadership Preparation models.^2^

### The Problem-Solving Cycle model

The original PSC is a research-based model of PD that focuses on teacher learning of mathematics content and instructional practices. This learning happens as teachers engage collaboratively in working on math tasks and discussing videos from math classrooms. PSC professional development is typically school-based and led by a teacher leader at the school. The model provides a focus and structure to school-based PD. It is a highly adaptable model that can be tailored to the goals, interests, and needs of participating districts and schools. This flexibility is key to affording districts and schools to take ownership of the PSC and make it relevant and responsive to their circumstances (Koellner et al. 2007; Borko et al. 2015).

**Fig. 1:**
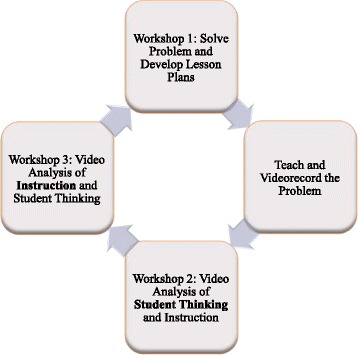
The Problem-Solving Cycle model of professional development

The PSC model consists of a series of three interconnected workshops organized around a rich mathematical problem referred to as the “PSC task” (Borko et al. 2015). Teachers solve the problem and prepare to teach it in their classrooms during workshop 1. They teach a lesson with the task between workshops 1 and 2, collecting video of both their instruction and students working on the task in small groups. They then use short video clips and student work from their PSC lessons to collaboratively analyze student reasoning and instructional practices in workshops 2 and 3 (see Fig. 1).

Participants engage in this cycle twice during the school year—once each semester. Typically, each cycle focuses on a different mathematical task and highlights specific issues related to teaching and learning. The structure of the PSC enables teachers to share a common mathematical and pedagogical experience and re-visit that shared experience via video, providing a foundation upon which to build a supportive community.

Video plays a central role in the PSC model, serving as a springboard for reflection and discussion about mathematics learning and teaching. Video has the unique ability to capture the richness and complexity of classroom life for later reflection and analysis. Because it can be shared, it provides teachers with a window into their colleagues’ classrooms and thus supports collaborative, practice-based learning (Sherin 2004; Borko et al. 2008; Groschner et al. 2014; van Es 2012). As Brophy (2004) cautioned, to be an effective tool for teacher learning, video must be viewed with a clear purpose in mind. When used in professional development programs, video must be purposefully selected to address specific program goals, and discussions must be carefully planned and orchestrated to achieve those goals (Borko et al. 2014).

The PSC uses video from the classrooms of teachers participating in the PD program. Video from any teacher’s classroom situates professional development in the practice of teaching and is likely to stimulate productive discussions. However, several researchers have reported that teachers find video from their own classrooms and the classrooms of colleagues to be more motivating and to better support their learning than video from the classrooms of teachers they do not know (Seidel et al. 2011; Zhang et al. 2011). In the PSC model, clips from participating teachers’ lessons using the PSC task provide a familiar mathematical context that enables the teachers to deeply explore mathematics learning and teaching and to generate specific ideas about how to better meet the needs of their particular student population. In addition, using video from teachers’ own classrooms reduces the possibility of teachers dismissing what they see because “those kids are not the same as ours.” Using video from classrooms where the teachers know each other requires a moderate amount of trust to view the student clips and a high degree of trust to watch the teacher clips. To share their videos, teachers must feel that they are part of a safe and supportive professional community. This must be accounted for in the design of the professional development sessions.

### The Mathematics Leadership Preparation model

**Fig. 2:**
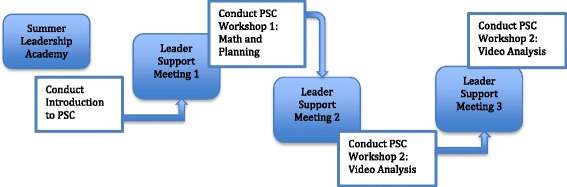
The Mathematics Leadership Preparation model

The original MLP model is designed to prepare PD facilitators, i.e., teacher leaders (TLs), to plan and lead PSC workshops. It consists of two components: a Summer Leadership Academy in which the TLs learn about the PSC and key facilitation practices for leading VBDs and Leader Support Meetings that provide structured guidance throughout the academic year as they prepare to lead PSC workshops (see Fig. 2), usually with colleagues at their sites. [Note: The PSC workshops are in the unshaded boxes and the Leader Support Meetings are in the shaded boxes. The alternation of these events gives a sense of how the two models are intertwined.]

During the Summer Leadership Academy, the TLs solve and analyze a variety of mathematics problems and select problems to use during the upcoming academic year. The leaders of the academy model the role of the TL as they introduce the mathematics problems and guide the analysis and discussion. The TLs also participate in a series of PSC simulations during which they plan and take turns leading the various activities that compose the three workshops. For example, they use classroom video from prior PSC cycles to plan VBDs, practice facilitating the discussions, and receive feedback from their peers and MLP leaders. In addition, they view video clips from prior PSC workshops and discuss the strengths and limitations of the facilitation practices. Finally, the TLs develop a general plan for implementing the PSC with the mathematics teachers in their schools during the upcoming year.

During the academic year, the TLs attend a Leader Support Meeting prior to each PSC workshop that they facilitate. Thus, for each cycle, there are three Leader Support Meetings. In these meetings, TLs receive structured guidance as they plan for their upcoming workshops. As in the Summer Leadership Academy, the TLs participate in typical PSC activities modeled by the MLP leaders. The meetings also include time for the TLs to work collaboratively to plan and rehearse workshop activities and to receive feedback from one another and the MLP leaders.

Throughout the MLP model, the TLs are learning the practices for planning and orchestrating a VBD. The approach that is used in preparing the TLs to facilitate a VBD was derived from the five practices for orchestrating productive mathematics discussions developed by Stein, Smith, and their colleagues (Stein et al. 2008; Smith and Stein 2011). These practices are (1) anticipating likely student responses, (2) monitoring students’ work, (3) selecting students to present their mathematical work during discussions, (4) sequencing the student responses, and (5) connecting students’ responses. Elliott et al. (2009) proposed that these five practices, understood somewhat flexibly, offer a useful model for teacher educators and PD facilitators. They suggested that by applying these practices in their work with teachers, PD facilitators could be more intentional in leading conversations around mathematics tasks.

In their chapter on facilitating VBDs, Borko, Jacobs, and colleagues (2014) argued that facilitators could use similar practices to engage teachers in productive conversations around video. They identified six elements that are related to the five practices listed above—three for planning a video-based discussion and three for orchestrating the discussion. The key elements for planning a video-based discussion are (1) determining the goals for the discussion and selecting video clips, (2) identifying features of the video clip that are important for meeting the goals, and (3) crafting questions to guide the discussion. The key elements for orchestrating the discussion are (1) eliciting teachers’ thinking about the lesson segment, (2) probing for evidence, and (3) helping the group to connect their analyses to key mathematical and pedagogical ideas (Borko et al. 2014). We incorporated these six elements in our approach to preparing TLs to lead VBDs.

As these brief descriptions illustrate, the MLP model provides multiple opportunities for participants to experience PSC activities modeled by the MLP leaders and to plan, rehearse, and receive feedback on their PD facilitation practices. In designing these experiences, we drew from Grossman and colleagues’ analysis of “approximations of practice”—“opportunities to rehearse and develop discrete components of complex practices in settings of reduced complexity” (Grossman and McDonald 2008, p. 190). When teachers are taking up a new teaching practice, including teaching one’s peers, they benefit from opportunities to try out the practice in a low-stake setting. Through the use of approximations of practice, or rehearsals, the teacher leaders are able to deliberately practice a potentially difficult part of the workshop they plan to lead with their math department colleagues. The rehearsal is followed by a brief discussion in which the TLs receive feedback from the others in the rehearsal.

In summary, there are three core activities in the PSC model that focus on the work of teachers:

- Working together as a group on a designated common math task
- Using the designated math task with their students with at least one teacher recording the lesson on video
- Participating in two VBDs—one with a focus on student learning and another focused on instruction

The key components of the MLP model build on the core activities of the PSC model to develop teacher leaders’ capacity to lead their peers through the PSC. They include the following:

- Collaboratively working on a math task and engaging in VBDs led by the MLP leaders
- Reflecting on each of the core activities of the PSC from the lens of the facilitators
- Planning two cycles of the three-part PSC workshop series
- Rehearsing difficult pieces of their workshop plans and receiving feedback

### Using a design-based implementation research approach to a research-practice partnership

The Stanford University researchers (herein referred to as the Stanford team) first approached the University/School District Partnership Coordinator and Urban Unified School District (UUSD) STEM leaders in the summer of 2013 to discuss the possibility of submitting a proposal to the National Science Foundation (NSF) to build district capacity to conduct mathematics professional development using adapted versions of the PSC and MLP models. Although the models provide a structure for professional learning experiences, they are also intentionally designed to be flexible and adaptable to local contexts. With this in mind, we suggested a design-based implementation research project in which we would collaborate with UUSD to adapt the models to align with district needs and priorities. The UUSD STEM leaders thought that the project would fit well with their current work to develop and implement a CCSS-aligned middle school math curriculum for the district. We worked collaboratively on the proposal, which was submitted to NSF in December 2013 and funded in January 2015.

This paper focuses on the initial DBIR cycle of design, implementation, and revision, which we refer to as the Design Phase of the project (spring 2015 to spring 2016) (Penuel et al. 2011). During that time, we worked with UUSD STEM leaders and the partnership coordinator to revise the models, implement the models at two school sites, and further revise the model components on the basis of our ongoing analysis of data collected during TLP sessions, PSC workshops, interviews with the TLs, and the TLs’ mathematics classes. One of our initial adaptations was to change our terminology to be compatible with language used in ongoing UUSD PD programs and with the evolving nature of our work. For example, we now refer to the model for preparing TLs as the TLP model.^3^

In the sections that follow, we describe the major adaptations we made to the models during the Design Phase of our project and the reasons for those changes. We begin with a description of the local context and the design and methods of the research project. We then elaborate on the adaptations, focusing primarily on adaptations that affected the nature of the VBDs.

### Local context

UUSD is a large urban district with high proportions of students from non-dominant cultural and linguistic communities (83% students of color, 25% English learners, and 60% students who qualify for free and reduced lunch). As such, this district provides an important setting for studying math instructional practices that meet the needs of diverse learners as well as all the complexity of an urban district with a commitment to high-quality math learning for all students (UUSD 2016). In 2015–2016, UUSD employed 3292 teachers with an average of 11 years of experience. This group includes 251 Nationally Board-Certified Teachers and 363 bilingual classroom teachers. Demographically, the teacher population is almost as diverse as the student population with one third of the teachers identifying as white, one fourth as Asian-Pacific Islander, one sixth as African American, and the rest declining to state or marking “other.” (UUSD 2016).

To align the UUSD middle school math courses to the CCSS, UUSD designed and developed a task-based curriculum for use in grades 6 through 8. Each curriculum unit follows the intentional sequence of an entry task, apprentice task, expert task, and milestone task. When working on these tasks, students have the opportunity to demonstrate their understanding of the essential concepts and standards in the unit. Within each unit, there are also interim lesson series to develop the students’ understanding of the specific concepts addressed in the tasks. Beginning in the 2014–2015 academic year, all middle school math teachers were expected to use the newly developed curriculum units. To assist in introducing the new curriculum to the teachers, UUSD identified teacher leaders who would participate in professional development workshops to understand the curriculum and then be responsible for bringing this knowledge back to their school sites.

We launched the Design Phase of the project by working with two middle schools selected by UUSD STEM leaders. Some teachers in these two schools had tried parts of the district’s new math curriculum, but neither school’s mathematics department was fully invested in an implementation strategy. This put them in a potentially receptive position for trying the PSC/TLP models. Working with the UUSD STEM leaders and each school’s principals, we identified the teachers to invite as teacher leaders on the project. Our goal was to recruit one math teacher per grade level at each site for a total of six teachers. District personnel identified three teachers from one school—two who taught sixth grade (one of whom was the math department chair) and one who taught eighth grade. At the second school, we started with three participating teachers—two sixth grade teachers, one of whom was the instructional lead for English learner (EL) instruction at the school, and an eighth grade teacher who was also the math department chair. Shortly after the Summer Institute, one of the sixth grade teachers from the second school was assigned other duties and did not continue on the project, so we worked with a total of five teachers during the first year.

None of the teachers in this initial group had experience leading professional development, and most were not very familiar with the district’s new math curriculum or the additional tools the district math team had developed to support the implementation of the curriculum. In addition, each school had distinct situations that complicated the curriculum implementation, as well as the integration of the PSC/TLP models, including the following:

- Changes in school leadership at both schools from AY 14–15 (recruitment) to AY 15–16 (launch). Both schools started the 15–16 academic year with new principals. One of the principals was new to the district; the other had been a teacher in the district.
- A major remodel at one school required the teachers to relocate their classrooms during the year—sometimes in the middle of the day. This increased their challenges for teaching task-based mathematics and also meant we could not easily conduct PD sessions on site.
- The other school has a “newcomers pathway,” which means this school serves many students who have recently entered the USA, resulting in a regularly changing student population at the school and within classrooms

### Project design

During the Design Phase of the project, we worked collaboratively with UUSD district personnel to modify the PSC and TLP models and the resources needed to support TLs’ facilitation of the PSC at their school sites using an iterative process of design, implementation, analysis, and revision (Cobb et al. 2003; Design-based Research Collective 2003; Penuel et al. 2012). Our primary objectives during this phase were to refine the PSC and TLP models and tools to reflect the goals of the district and to fit the needs of the two Design Phase schools, to integrate key ideas from the district’s program to address the educational needs of linguistically diverse student populations, and to prepare the tools and resources for use in the phase two comparison study.

In addition to being involved in the project design at the study’s outset, UUSD district personnel have been directly involved in the planning of all PD activities for the TLs. During the summer, the Stanford team worked with the UUSD STEM leaders as a collaborative group we refer to as the “Design Team.” The Design Team developed an agenda for the Summer Institute that coordinated with other summer PD offered in the district for middle school math leaders. Our intent was to make sure that the TLs in the PSC would not only begin learning the PSC model but would also learn and experience what the other TLs in the district were experiencing. Therefore, our agenda during the summer needed to provide opportunities for teachers to go through the revised curriculum, noting the resources available to the teachers and parents, and the toolkit with its various instructional strategies that teachers are expected to implement in the classroom. These district priorities for the Summer Institute substantially reduced the amount of time devoted to the PSC compared to the original PSC/TLP design.

In summer 2015, the six designated TLs participated in a 3-day Summer Institute in which they learned the PSC model and prepared for their first school-site PD session. Specifically, the TLs solved and analyzed the math tasks they would use in the first PSC cycle, participated in a VBD, and planned their initial workshops to introduce the curriculum and the PSC to the teachers in their schools, but they did not engage in rehearsing any components of the PSC workshops. In addition, we introduced the TLs to a video recording application for use on tablets or smart phones and its associated online platform. They had time to practice using these tools to design an introduction to the PSC and the new math curriculum so they could lead the first workshop with math teachers during the teacher in-service days before school started.

During the 2015–2016 academic year, the Design Team led the TLP sessions and supported the TLs as they facilitated two iterations of the PSC at their school sites. The TLs attended six all-day TLP sessions during which they planned all aspects of the PSC workshops and rehearsed key aspects that they were concerned about.

## Methods

In this section, we describe the data sources we drew upon and analyses we conducted to answer the two research questions that were central to the Design Phase of this design-based implementation research: how we adapted the PSC and TLP models to support the district goals for implementing their new CCSS mathematics curriculum and conditions that shaped the adaptations. As noted above, in keeping with the theme of this special issue, we highlight the adaptations related to the use of VBDs and the preparation of TLs to use VBDs at their sites.

### Data sources

The primary data sources for the analyses reported in this paper include:

- Artifacts from professional development sessions for the TLs (the Summer Institute and six TLP sessions) from August 2015 through April 2016, including detailed agendas and PowerPoint slides
- Field notes focused on changes to the planned professional development sessions
- Research memos written by Stanford team members each time they interacted with someone from UUSD
- Emails between the Stanford team and the UUSD STEM leadership
- Minutes of weekly project meetings
- Transcripts of interviews conducted with the TLs before and after the Summer Institute and after PSC 1 and PSC 2.

### Data analysis

We relied primarily on the agendas, slides, and field notes from the Summer Institute and TLP sessions to identify changes to the PSC and TLP models. We compared the field notes to the agendas and slides for each session to create a list of all changes that represented adaptations to the PSC or TLP models. In addition, minutes of our weekly project meetings provided a record of key decisions about changes in the goals, activities, and intended outcomes for TLP sessions and reasons for those changes.

To analyze these data, we started with a set of four a priori codes identified as possibly pertinent to the implementation of the models based on district priorities and structures as well as recurrent themes in the literature on school and district change (Fullan 2007; Sarason 1996). Each data source was qualitatively coded using the four categories: multiple roles for TLs as teachers and as instructional leaders, school dynamics, district leadership goals and characteristics, and unique district characteristics (e.g., new curriculum, school demographics, teachers, other simultaneous initiatives). We were also open to including other categories that may have emerged from our review of the data; therefore, we added an “Other” category.

We worked in pairs to code changes and adaptations according to those categories and attempted to sort the changes identified from each data source into categories. To do this, we created a data display with excerpts from the data sources representing the a priori codes (Miles et al. 2014). During this meeting, we reviewed the ideas that surfaced within smaller teams and checked for understanding as well as consistency of labeling. We found that coding our data this way did not reveal any patterns with explanatory power.

When this initial analysis did not yield meaningful results that we could relate to the adaptations we had made, we began to test ideas we saw emerging by iteratively reviewing the themes and evidence for them. We focused on themes related to the development of and changes to video-based discussions (VBD) in this round of analysis. This iterative process continued until we had checked and re-checked each line of evidence against the emergent themes and found that all the evidence had a thematic home. We realized that the nature of these adaptations was more related to district “givens” and the limited experiences TLs had in a variety of categories. As a consequence of seeing these patterns, we reorganized our data based on emergent themes that had more explanatory power regarding the adaptations that we made to the models. We then analyzed the two categories of changes to the models and the related decisions to identify patterns in the adaptations and reasons for the adaptations.

We used our observations of the TLs during TLP sessions, the TLs’ reflections written and discussed during TLP sessions, and their comments in the interviews to identify patterns in their experiences and thoughts about being a TL and the nature of their interactions as a site-based team. We also used the end-of-year interviews with the TLs to identify recurring or unique comments related to their impressions of the VBDs or the impact that experiences such as participating in VBDs, rehearsing VBDs, and leading VBDs during PSC workshops had on their development as teacher leaders. Any thought or observation documented by a researcher as well as direct statements made by teachers that captured a TL’s experience of being a TL was considered a piece of evidence. We culled the pieces of evidence by individual TLs to begin to characterize the experience of being a TL. We also looked across the sets of evidence for individual TLs to identify patterns that applied to multiple TLs.

This work of identifying patterns across data sets was conducted by subgroups within the Stanford team. When these analyses were completed, the Stanford team met to review the patterns that emerged regarding adaptations to the PSC and TLP models and development of the teachers as teacher leaders. We then compared the two sets of patterns to determine the relationship between the changes in the models and the TLs’ development and to identify the ways in which we adjusted implementation of the PSC and TLP models to support both the TLs’ development and district priorities.

## Results and discussion 

In this section, we describe the two categories of adaptations that we made to the models from the perspective of supporting the TLs to participate in and lead VBDs:

- Adaptations to address district priorities
- Adaptations to address the limited experience of the teacher leaders

### Adaptations to address district priorities

As indicated above, UUSD had developed a new middle school math curriculum that we needed to incorporate into our work. In addition, they had developed an explicit vision for mathematics for all students in the district that specifically included English learners. These “givens” were not part of the original models but were also not antithetical to the models. Given the DBIR approach of the research-practice partnership, we were able to work with the district STEM team to adapt the models to reflect district priorities.

### Adapting the PSC to the new UUSD curriculum

During the process of designing the project and writing the proposal, the Stanford/UUSD Design Team agreed that the PSC tasks would be tasks from the new curriculum units and that rather than all teaching the same task, teachers would only teach tasks that were part of their grade-level curriculum. We would therefore have three PSC tasks per cycle, one at each grade level. A key feature of the original PSC model is that all teachers share a common mathematical and pedagogical experience so that when they examine video in workshops 2 and 3, all would have analyzed and taught the PSC task used in the video clip. Thus, this decision represented a major adaptation to the original design.

To operationalize this decision, the Stanford team identified units for each cycle at the sixth, seventh, and eighth grade levels that addressed the same content strand and would be taught relatively early in the semester. In cycle 1, we chose proportional reasoning, and in cycle 2, we chose statistics and data representation. From these units, we chose tasks that met our criteria that they were “group worthy” and could be used to highlight strategies for addressing high language-demand math tasks.

In addition to the constraints placed on task selection, this adaptation created several challenges for the Stanford team to address in order to integrate the three grade-level tasks into the first TLP session and subsequent PSC workshop. Even though the units addressed the same general key concepts, within each unit, the content standards were different. As a result, we anticipated that we would need to allocate time in the TLP session to explore the mathematical content of all three tasks.

### Addressing the needs of English learners

The development and implementation of the UUSD math curriculum was occurring concurrently with a newly adopted policy for de-tracking students in their math courses and a commitment to universal access, particularly for English learners. Therefore, the district requested that the models be adapted to specifically and explicitly address the needs of English learners. From the TLs’ comments during interviews, it appeared that some teacher complaints about the new curriculum were fueled by a sense that the curriculum was difficult to teach, especially to ELs, because of its emphasis on language-rich text. Joy noted, “because it’s like you have a word problem with every page and they don’t understand these things.” Teachers were also not certain of the relevance of the curriculum to their student population. In her pre-summer interview, Lynn shared, “It’s so different than what it was. … You know, adjustments. So I need to see if there’s anything that doesn’t really transfer well. How can I take this and make it relate to something from another culture?”

Drawing on the flexibility of the PSC and TLP models, we were able to incorporate several tools and activities to address these UUSD priorities. We created a Universal Access (UA) Framework to support equitable access to mathematics for all students. This framework is built on Goldenberg’s (2013) review of research relating to effective instruction for English learners and the TRU Framework, which the district had already adopted and adapted (Schoenfeld 2014). The UA Framework is a list of categorized questions for teachers to use when collaboratively planning or reflecting on instructional practice and student access.

We introduced the UA Framework to TLs during the Summer Institute. They used the framework as a thinking frame as they reviewed the introductory unit from their curriculum. They looked for ways that strategies for universal access were already present in the curriculum. We revisited the UA Framework in TLP 2–1 (the first TLP session in the second cycle) after the TLs raised particular concerns about meeting the needs of English learners in their classrooms. In TLP 2–2, we built on this work by focusing on effective questioning as an instructional practice to support universal access. In addition, in response to a request from the district, we leveraged existing district resources in conjunction with the UA Framework. Specifically, we incorporated pieces of the toolkit the district had assembled to support the implementation of the math curriculum. For example, we modeled the use of participation quizzes and effective questioning strategies.

In addition to generating and relying on the UA Framework as a source for thinking about improving the math learning experience for English learners, the Stanford team consciously chose video clips that illustrated times when student voices were roughly equal regardless of the primary language of the learner, as well as times when some students were silenced.

We also developed questions to guide VBDs in ways that supported the teachers in considering how to provide universal access to mathematics with a particular emphasis on strategies for teaching math so that students develop agency, authority, and identity in mathematics. This approach to providing universal access was a direct reflection of the district’s priorities about the nature of the learner’s experience in the mathematics classroom. An example of how we addressed the agency, authority, and identity aspect of universal access can be seen in TLP 2–3 in which we conducted a VBD guided by these questions:

- Where do you see a moment when a student is “in charge” of the mathematics?
- What evidence do you see of opportunities that foster students’ willingness to engage with the mathematics?
- What are the teaching moves that enhance student willingness to make their reasoning public?

By providing this emphasis, we reinforced the district’s goal of inclusivity with particular attention to English learners who have had less agency, authority, and identity in the mathematics classroom.

### Adaptations to address the limited experience of the TLs

The TLs with whom we worked had little familiarity with the new curriculum or with leading professional development. The majority of the TLs indicated that they had not taught much of the new curriculum in the 2014–2015 school year. Further, in the summer interviews, all of the TLs expressed unease with using the new curriculum and a sense that it was difficult to teach. In particular, they were uncomfortable with the nature of the group work that was emphasized in the curriculum. Hearing their concerns, we realized that the TLs needed to prepare for teaching the PSC tasks to their students as well as for leading workshops about the tasks with their colleagues.

In addition, due to the timing of recruitment, the TLs were not familiar with the PSC prior to becoming PSC teacher leaders. We initially planned that the TLs would participate in the PSC as teachers during spring 2015 so that when we conducted the Summer Institute, they would have had first-hand experience of how the PSC model works from the perspective of teacher learners, and would have developed as a professional learning community. Then, in the Summer Institute, they would have been able to reflect on their own learning and also watch video from the PSC workshops in which they had participated to analyze and reflect on the Stanford team’s facilitation practices as they began to think about their new roles as PSC teacher leaders. However, because the project launched in January 2015 rather than September 2014, we were not able to recruit the TLs in time for them to participate in the PSC as teachers during spring 2015. Instead, they learned the PSC model as they also learned to be TLs and lead their colleagues through two PSC iterations.

These limitations in the TLs’ experiences had a significant impact on our plans to implement both the TLP and PSC models. In this subsection, we unpack how we adapted the models to address them.

### Increasing the emphasis on being an instructional leader

Although two of the five TLs were department chairs during their participation in the Design Phase, we quickly discovered that their department work focused much more on administrative tasks than instructional leadership. None of the TLs had done any work leading their peers in an examination of the new mathematics curriculum, student thinking, or instructional practice. Further, some TLs were unfamiliar with facilitating activities with adult learners, which made the TL role more challenging. These challenges were also aggravated by their lack of familiarity with the curriculum, which was the focus of their collaborative inquiry.

Throughout the Stanford team’s planning and implementation of the TLP meetings, we included activities in which TLs engaged as teachers and as teacher leaders. To help the TLs differentiate between learning experiences related to these two roles, we adopted the use of explicit language to identify when they were in “teacher” mode and when they were in “teacher leader” mode. We kept these activities distinct and identified them explicitly by noting when they were changing “hats.” For example, all VBDs during the TLP meetings were designed to include two components. First, TLs engaged as teachers by watching the video and discussing what they noticed and learned about the mathematics, learning, and teaching. Next, we asked the TLs to switch to their teacher leader hats, and we led them through a debrief of the facilitators’ moves. This approach also helped us embed the PSC work for the TLs as teachers without having them be confused by whether or not they were engaged in an activity as a teacher (PSC) or as a teacher leader (TLP).

### Strengthening the role of modeling and debriefing activities to support leadership development

Although modeling activities were an explicit part of the original TLP model, debriefing activities were not. One adaptation we made in response to the TLs’ lack of familiarity with the new curriculum, the PSC, and peer instructional leadership more generally was to incorporate both modeling and debriefing into each of the TLP sessions. Our use of modeling and debriefing evolved over the course of the Design Phase year, based on how the TLs responded during TLP sessions, our conversations with the UUSD members of the Design Team, and our own reflections captured in research memos. In this section, we describe this evolution chronologically, from the first TLP session in cycle 1 of the Design Phase (TLP 1–1) through the final session in cycle 2 (TLP 2–3). The evolution includes the addition of debriefs, the use of a specific protocol to guide debriefs, the use of video from a Stanford team member teaching middle school math for early VBDs, and a change in timing when we introduced a focus on instruction during VBDs to support deeper analysis of student learning.

In TLP 1–1, we modeled PSC workshop 1 using a seventh grade task. As we had done in the original TLP model, we used a “Teacher Analysis Task”^4^ that focused on possible correct and incorrect student solution strategies. After one member of the Stanford team modeled the use of that task with the TLs, we debriefed the activity by asking the TLs to jot down notes on a protocol that included the following questions:

- What goals did the teacher leader appear to have?
- What strategies did the teacher leader use to promote a deeper understanding of the mathematical concepts?
- What strategies did the teacher leader use to unpack potential stumbling blocks?

We then facilitated a discussion in which the TLs shared their observations and insights about the teacher leader’s role based on the notes they recorded in response to the questions.

Leading a VBD was new for all the TLs. We therefore decided to conduct very detailed debriefs of the Stanford team members who led the discussions. This allowed us to make the preparation that goes into a high-quality VBD explicit. In anticipation of not being able to have access to video of teachers using the math tasks from the curriculum during cycle 1, a Stanford team member taught and recorded a lesson using the eighth grade PSC task in a summer school setting during July 2015. We used video clips from his teaching to model VBDs in TLPs 1–2 and 1–3.

The clip we used in TLP 1–2 focused on Anthony’s launch of the math task. Two other members of the team led the VBD, which focused on identifying evidence in the clip that students understood or did not understand the task.

Following the discussion, another member of the Stanford team led the debrief of the facilitator. Her questions included:

- What were your goals for the VBD?
- Why did you select this clip?
- What did you want the teachers to notice? To discuss?
- Did you have back-pocket questions to ask? What were some of them?
- Why did you choose to show the clip 3 times?
- Why did you show it without a prompt the first time?
- Was there anything unexpected that came up in the conversation?

Given some Stanford team members’ observations in their research memos that the TLs did not delve deeply into student learning during their VBDs and the comments by teachers that they were not sure they wanted to share videos of themselves teaching with their peers, we did not shift to a focus on instruction for the VBD and debrief in TLP 1–3. Instead, we continued to focus on student thinking, this time examining interactions during group work. This decision represented another adaptation of the TLP and PSC models.

For TLP 1–3, we modeled the VBD with a clip of Anthony’s summer school class focused on a group of four students working on and discussing the math task. He co-facilitated the discussion of his own video with another member of the Stanford team. After the group watched the video twice, he launched the discussion by asking, “What evidence do we have of each student’s understanding of the math?” After a few minutes, the co-facilitators handed out a transcript of the clip. Their questions then further probed for evidence of the students’ understanding. The facilitators closed the discussion by asking the group what insights they gained about student understanding from watching the video.

A third member of the team conducted the debrief of the VBD. She began by asking the facilitators what they wanted the teachers to notice in the video and what types of questions they had prepared to address that goal. A fourth member charted the co-facilitators’ responses to the debrief questions. After the debrief, she turned the TLs’ attention to the chart and asked: “What insights did you gain about planning and conducting VBDs by listening to the two co-facilitators debrief?” This combination of the debrief and discussion of the charted responses was quite detailed, in order to make the planning that goes into a high-quality VBD explicit for the TLs.

This explicit attention to making our planning transparent resulted in the TLs commenting that they had not realized how much time and preparation we were putting into the VBDs we led with them. In particular, they were surprised to see how specifically we identified our goal for the discussion and details in the clip that we wanted them to notice, the time we spent determining possible teacher responses, and how we prepared numerous “back-pocket questions” to guide the discussion and keep it on track. As a result of this experience, they began to understand the amount of detailed planning they needed to put into the VBDs they were going to lead with their peers on site.

We continued to refine the modeling and debriefing activities as we began the second cycle during the spring semester. Because of the TLs’ and teachers’ lack of familiarity with the curriculum, we decided to abandon the Teacher Analysis Task in TLP 2–1 and PSC workshop 2–1 and have both the TLP session and PSC workshop focus on analyzing the math content and practices. We also decided to model using just the sixth and eighth grade tasks, which we were able to do because an UUSD STEM instructional coach agreed to support the seventh grade teachers during the site-based PSC workshops at each school.

We began TLP 2–1 with one member of the Stanford team modeling “Doing the Math” with the eighth grade TLs and another modeling with the sixth grade TLs. The two Stanford team members planned the activity together and used a parallel structure with both groups of TLs. We video-recorded these activities. Then, while the TLs were working on an activity related to universal access, we identified a 12-min clip of the Stanford team member facilitating the eighth grade “Doing the Math” activity for the TLs to watch during lunch with these two questions in mind:

1. What do you think Anthony’s goal was in leading this discussion of the task?
2. What evidence do you see to support that goal?

After the TLs viewed the clip, we discussed what they thought the goal of the discussion had been and what evidence supported their responses. Then another member of the Stanford team debriefed Anthony, focusing her questions on the goals of the activity, how he planned the activity, his use of a chart to guide the discussion, and specific facilitation moves that she had noted during the activity. These modifications enabled the TLs to learn to lead workshop 2–1 while focusing closely on their own grade-level curricula, so that they felt better prepared to both teach the task to their students and facilitate that workshop with their peers.

The structure of the modeling and debriefing activities for TLP 2–2 was similar to that of TLP 1–2, with two members of the Stanford team co-facilitating the VBD and a third one debriefing. The major change was that we used video of a small group of students in one of the TL’s classrooms working on the sixth grade PSC task. We decided that the group of TLs had developed as a professional learning community with enough trust and support to watch and discuss video of one of their classrooms. Because we were using video of UUSD students working on the task, we were able to focus specifically on the TLs’ students’ understanding of the UUSD curriculum tasks and implications for next steps in their teaching. The VBD concluded with the question, “Given that this is an Apprentice Task, how does what the students say inform your planning as you prepare the students for the Expert Task?”

We also co-facilitated all of the VBDs as an explicit form of modeling how the teams of TLs from each school site might work together to lead their VBDs. Understanding that co-facilitation often involves subtle moves that might be missed by novices, we took time in the debriefs to unpack the roles of each facilitator. To illuminate ways of approaching co-facilitation, we asked the facilitation team questions such as the following: How did you organize your co-facilitation? How did you plan for co-facilitation? Was there anything the other person did that surprised you?

### Scaffolding the selection of video clips

Selecting short and effective video clips from full-class videos can be a time-consuming and somewhat daunting task. The original TLP model includes tools to scaffold and streamline this task, such as a video selection matrix. The matrix describes the types of clips that are likely to be effective for PSC workshops 2 and 3, organized by where the situation depicted in the clip typically would occur in a lesson. For example, for workshop 2, the matrix identifies situations likely to be found during group work such as students debating two or more different solutions to the task. For workshop 3, it identifies situations during the closure of a lesson such as the teacher facilitating a class discussion about students’ solutions.

Given the TLs’ limited experience with the PSC and instructional leadership more generally, as well as the limited amount of time they had to select a clip and then plan and rehearse their VBDs, we decided not to introduce the video-selection matrix and instead to scaffold the task to an even greater extent. Prior to TLP 1–2, members of the Stanford team selected three 15-min clips from the hours of classroom footage each school had to work with. This narrow focus helped keep the task of choosing a 2- to 3-min clip for use in their PSC workshops more manageable. After the first cycle, we streamlined this process even further by only giving the TLs one 15-min video clip to watch in order to select their 3-min clip. This degree of scaffolding enabled us to guide the TLs to select clips during portions of the lesson when students were engaged with the PSC task and that featured students’ mathematical thinking. In addition, it enabled the TLs to have more time to plan and rehearse their VBD.

### Using rehearsals and debriefing activities to support leadership development

Rehearsals were part of the original TLP model. However, the model included rehearsals of VBDs, not facilitation of the math tasks. Because the curriculum was so new to these TLs, as was the approach of teaching with tasks, we added rehearsals of “Doing the Math” in both TLP 1–1 and TLP 2–1.

We realized after the first TLP meetings that the TLs were so unfamiliar and uncomfortable with the idea of rehearsing in front of their peers and the Stanford team that we needed to add more support and scaffolds. Their initial “rehearsals” were more like walk-throughs than true rehearsals; that is, they talked through what they would do rather than actually rehearsing their facilitation. Beginning with TLP 1–2, we changed our preparation instructions and worked very closely with the teams to plan their rehearsals. A subset of the Stanford team sat with each team during their planning time and helped them to select the video clips and plan launching and back-pocket questions. We also helped the teams decide on their co-facilitation roles because we noticed an uneven distribution of labor.

Each rehearsal was debriefed in a discussion facilitated by a member of the Stanford team. These debriefs were very different from the debriefs of the modeling activities. Here, the intention was for the participants in the activity to provide feedback to the TLs and for the TLs to learn from one another’s rehearsals as well as their own. We used a Praise-Question-Polish (PQP) protocol (see Table 1). PQP is a strategy for peer feedback designed to guide participants to provide specific, constructive feedback to their peers. It is used in a variety of activities including writing assignments, oral presentations, and, in this situation, rehearsals (Neubert and McNelis 1990). Prompted by sentence starters in the protocol, participants in the activity—the Stanford team, other TLs, and UUSD district personnel—first offer praise for positive aspects of the activity. They next ask questions about any aspects they found confusing and then offer suggestions about aspects that could be polished or revised. This form of debrief provided a supportive structure within which the TLs could receive constructive feedback and hone their skills for facilitating effective VBDs.

**Table 1:** Praise-Question-Polish protocol

• Praise	
I appreciate how you…	
When you…it really…	
I never thought about … and when you … it inspired me to think about	
• Clarifying questions	
What was the intention of…	
What did you mean by…	
What was going through your mind when…	
• Points of polish	
Have you thought about…?	
Might you consider…?	
I think maybe if you….	

## Conclusions

The structure of the PSC enables teachers to share a common mathematical and pedagogical experience and re-visit that shared experience via video, providing a foundation upon which to build a supportive community. Because video clips from participating teachers’ PSC lessons are such an integral part of PSC workshops, a central focus of the TLP sessions is to provide opportunities for the TLs to plan and rehearse VBDs. During the development of the PSC and TLP models, a significant portion of time was devoted to TLs looking through the available video and identifying clips likely to foster rich conversations about student reasoning and instructional practices, with the assistance of the TLP leaders. Once these clips have been identified, the Math leaders plan both launch questions and “back-pocket” questions to guide the discussions and then rehearse the discussions with the group.

As described in the “Results” section, in our current project, we adapted the PSC model during the Design Phase to reflect recent policy changes in the district as well as their very explicit vision that “All students will make sense of rigorous mathematics in ways that are creative, interactive, and relevant in heterogeneous classrooms.” The policy changes included the development and implementation of the new math curriculum, explicit attention to English learners, and a focus on creating the opportunity for all students to develop the capacity and willingness to engage with and show command of the content (UUSD 2016). We were also working with teacher leaders who had extremely limited experience with the new district curriculum, the PSC, and/or leading professional development of any type. As the following comment from one TL’s post-summer interview indicates participating in the PSC/TLP work had a positive impact. She shared that before engaging in PSC professional development, her department typically “spent a lot of time complaining about the Common Core.” This year, in comparison, the department meetings are focused on “how do we really make this work for our students?”

We made adaptations to the TLP and PSC models in response to the priorities of our district partner and the specific needs of our TLs and their sites. District priorities drove the changes that included incorporating math tasks from the UUSD curriculum at all three grade levels and focusing significant attention to the needs of ELs. Among the most notable adaptations are those associated with the timing of the TLs’ experience with the PSC as teacher-learners, their lack of familiarity with the district math curriculum, and their newness to the role of being a peer instructional leader. In response to these needs, we significantly increased the role of modeling and debriefing to provide more learning opportunities for the TLs during the TLP sessions. We further strengthened modeling and debriefing through the inclusion of rehearsals and PQP debrief protocols. We also supported the TLs’ learning by providing more extensive scaffolds as they selected video clips, designed and rehearsed their workshops, and debriefed their VBDs. This set of activities formed an iterative cycle of learning new practices. The activities extend Grossman and colleagues’ framework for the teaching of complex professional practice to preservice teachers to the work of professional development facilitators. Our adaptations provide powerful examples of the framework’s three pedagogies of practice in this new context: representations (modeling of PD activities), decompositions (debriefs of the modeled practices), and approximations of practice (rehearsals and accompanying debriefs) (Grossman et al. 2009). They thus contribute to the growing body of research on the power of these pedagogies as tools for supporting the learning of ambitious teaching practices (Kazemi et al. 2016; Lampert et al. 2013).

The key tenets of the PSC and TLP models include (1) supporting teachers to develop mathematical knowledge for teaching and enhance their repertoire of effective instructional strategies by doing math together, (2) engaging in video-based discussions of learning and teaching, and (3) building professional communities. Even with the multiple adaptations we made in the course of our DBIR work with UUSD, the current versions of the PSC and TLP models remain aligned to their theoretical roots and purpose of supporting effective math teaching and the development of professional development leaders. As we expand the project to include additional schools, we are encouraged by the progress the Design Phase TLs have made and their increased confidence as leaders. As one of the teacher leaders noted in her interview at the end of the school year: “What was really helpful was practicing, you know, and constantly being reminded like ‘What’s the question? What do you want them to get or see or talk about?’ And then, … questioning the teachers in the discussion to try to lead them towards answering that central question.”

Our experiences adapting and implementing the PSC and TLP models in UUSD have implications that extend beyond the specifics of the two models. These implications are grounded in our experiences from our research-practice partnership and include insights about the preliminary work to do before launching any professional development program that incorporates video-based discussions and key factors to attend to when planning and conducting the programs.

Coburn et al. (2013, p. 2) define research-practice partnerships at the district level as “Long-term, mutualistic collaborations between practitioners and researchers that are intentionally organized to investigate problems of practice and solutions for improving district outcomes.”

In this case, we are working with UUSD to support their vision of mathematics education by adapting a pair of established models for conducting site-based professional development and developing teacher leaders. We are using a DBIR approach in this work, building from the district’s vision and recognition of their persistent problems of practice during the Design Phase to design, implement, analyze, and revise the PSC and TLP models.

It has been critical that our work support the learning goals and priorities of the district (Coburn and Penuel 2016). Video can be a powerful tool for supporting teacher learning. PD facilitators can select video clips that depict features of learning or teaching that address district priorities, and they can develop launching and back-pocket questions that focus teachers’ attention and guide conversations to address those features. For example, one of the UUSD’s priorities was to ensure universal access to the mathematics content (UUSD 2016). We therefore modeled a VBD focused on the universal access framework and supported TLs to select video clips and plan and rehearse VBDs with a similar focus for their PSC workshops. In her interview at the end of the year, a district math coach highlighted the importance of video-based discussions, noting that they “helped illuminate what mathematical discourse could look like in a classroom,” which, she explained, “is new ground for many teachers.” Several of the TLs also commented about the value of watching and analyzing classroom video during the PSC workshops.

While the program includes developing the capacity of teacher leaders to conduct the PD, it is important to assess the extent of their leadership experience and skills and to take this information into account in designing preparation and support activities. Although video is being used more widely in practice-based professional development (van Es et al. 2014), it is likely that VBDs will be unfamiliar to many novice TLs. Our experiences working with UUSD point out the value of designing leadership preparation sessions that include time to model how to conduct a VBD, an opportunity to unpack the VBD by debriefing the facilitator(s) who modeled it, and then space for TLs to plan and rehearse a VBD with a specific protocol-based debrief that reduces anxiety and supports their growth. In their end-of-year interviews, several of the TLs commented on the value of these activities when asked which activities in the TLP sessions most helped them prepare to facilitate VBDs. One TL shared her thoughts about the value of the modeling and debriefing, “When [one of the Stanford team] would come in and debrief you about what you did and how it felt, it dawned on me pretty quickly that you were modeling what we were going to be doing. Your insights into what you were thinking, how it worked, what you were trying to get at with this activity and the line of questioning, I found it all very, very helpful.” Another explained that the rehearsals “gave us a chance to practice asking questions beforehand and got us thinking, ‘Is that the right question we wanted to ask? Did it work or did it not work?’ so it just gave us a chance to tweak what we needed to do....” Similar to novices preparing for professional practice in the clergy and clinical psychology, these novice TLs benefitted from the representations, decompositions, and approximations of practice we incorporated into the leadership preparation sessions (Grossman et al. 2009).

These suggestions are based on the experiences of a very small number of novice teacher leaders and their specific school settings in a district with a well-defined vision. We look forward to testing the adaptations further as we implement the revised PSC/TLP models with a larger group of schools in our phase two comparison study. During this next phase, we anticipate being able to offer findings that speak to the role of a research-practice partnership in building district capacity to support long-term change. We anticipate that the modifications and materials will be useful for other PD facilitators and facilitator educators who want to focus on the role of VBDs for improving teaching and learning and that other researchers will study the education of PD facilitators and the materials and activities that are effective in supporting their learning.

## Abbreviations

CCSS: Common Core State Standards
MLP: Mathematics Leadership Preparation
NSF: National Science Foundation
PD: Professional development
PSC: Problem-Solving Cycle
TLP: Teacher Leadership Preparation
TLs: Teacher leaders
TRU: Teaching for Robust Understanding
UA: Universal Access
UUSD: Urban Unified School District
VBD: Video-based discussion

## Footnotes

1 Note that the names of the district, schools, and teachers are psuedonyms. We use actual names of the Stanford team members.

2 The two models were developed and field-tested as part of the two research projects conducted by Borko, Jacobs, and Koellner, supported in part by grants from the National Science Foundation (Grant No. DRL 0115609; Grant No. DRL 0732212). For more detailed descriptions of these models and the research studies, see Borko et al. (2015).

3 We changed the name of the summer component from Summer Academy to Summer Institute in this project to match the language that UUSD uses for their summer program for teachers. The MLP model was changed to the Teacher Leadership Preparation (TLP) model. The leadership preparation “meetings” were renamed TLP “sessions” to capture the interactive nature of those all-day experiences, which were more similar to PD sessions than meetings. We also revised the term “MLP Leader” to “TLP Facilitator” to denote members of the Stanford team who were leading (or facilitating) the work during TLP sessions.

4 In developing the PSC model, we found that rather than having the teachers solve the PSC problems—which are designed for students—workshop 1 is most effective if the facilitator creates a Teacher Analysis Task. One type of Teacher Analysis Task that we found to be effective includes the PSC problem together with a variety of solution strategies (both correct and incorrect) that students are likely to use. The Teacher Analysis Task is to carefully analyze each solution strategy, thinking through which are correct or incorrect, why, and the potential gaps in students’ knowledge. By considering these questions, teachers can begin to anticipate how their own students might solve the problem (Borko et al. 2015).
